# Amygdala-liver signaling orchestrates rapid glycemic responses to stress and drives stress-induced metabolic dysfunction

**DOI:** 10.21203/rs.3.rs-2924278/v1

**Published:** 2024-03-29

**Authors:** Sarah Stanley, Kavya Devarakonda, Richard O’Connor, Maria Jimenez-Gonzalez, Alexandra Alvarsson, Rollie Hampton, Diego Espinoza, Rosemary Li, Abigail Shtekler, Kaetlyn Conner, Mitchell Bayne, Darline Garibay, Jessie Martin, Vanessa Lehmann, Liheng Wang, Paul Kenny

**Affiliations:** Icahn School of Medicine at Mount Sinai; Icahn School of Medicine at Mount Sinai; Icahn School of Medicine at Mount Sinai; Icahn School of Medicine at Mount Sinai; Icahn School of Medicine at Mount Sinai; Icahn School of Medicine at Mount Sinai; Icahn School of Medicine at Mount Sinai; Icahn School of Medicine at Mount Sinai; Icahn School of Medicine at Mount Sinai; Icahn School of Medicine at Mount Sinai; Cornell Univ; Icahn School of Medicine at Mount Sinai; Icahn School of Medicine at Mount Sinai; Icahn School of Medicine at Mount Sinai; Icahn School of Medicine at Mount Sinai

## Abstract

Behavioral adaptations to environmental threats are crucial for survival and necessitate rapid deployment of energy reserves. The amygdala coordinates behavioral adaptations to threats, but little is known about its involvement in underpinning metabolic adaptations. Here, we show that acute stress activates medial amygdala (MeA) neurons that innervate the ventromedial hypothalamus (MeAVMH neurons), which precipitates hyperglycemia and hypophagia. The glycemic actions of MeAVMH neurons occur independent of adrenal or pancreatic glucoregulatory hormones. Instead, using whole-body virus tracing, we identify a polysynaptic connection from MeA to the liver, which promotes the rapid synthesis of glucose by hepatic gluconeogenesis. Repeated stress exposure disrupts MeA control of blood glucose and appetite, resulting in diabetes-like dysregulation of glucose homeostasis and weight gain. Our findings reveal a novel amygdala-liver axis that regulates rapid glycemic adaptations to stress and links recurrent stress to metabolic dysfunction.

## Introduction

When an animal encounters a threat in the environment or other stressful stimuli, it must rapidly mobilize energy stores to support appropriate defensive behaviors, such as escape, darting, or freezing^1–3^. Rapid energy mobilization fuels cardiovascular and muscular responses to stress and supports the increased allocation of cognitive resources necessary to process threat-relevant contextual information^4–6^. In parallel, behavioral repertoires that could compete with defensive strategies, such as foraging^7^ and eating^7^, must be suppressed. Glycemic responses to stress are correlated with fight-flight behaviors^8^, and glucose infusions elicit stress-like enhancements of cardiac output and blood pressure^9–12^, and facilitate the encoding of spatial memories^13,14^. These threat-induced metabolic adaptations are highly conserved across species, suggesting they provide a strong evolutionary advantage^15^. Despite their biological importance, surprisingly little is known about the brain circuits that orchestrate adaptive hyperglycemic and hypophagic responses to acute stress^16^.

Here, we identify a novel amygdalar-hypothalamic-liver axis that regulates rapid metabolic adaptations to acute stress via mechanisms independent of the classical adrenomedullary and hypothalamic-pituitary-adrenal (HPA) stress systems. We demonstrate that recurrent stress disrupts amygdalar-liver circuit activity to drive stress-related metabolic abnormalities, thereby linking chronic stress to metabolic disorders such as type 2 diabetes (T2D) and obesity.

## Acute stressors mobilize rapid metabolic adaptations

**Figure 1:**
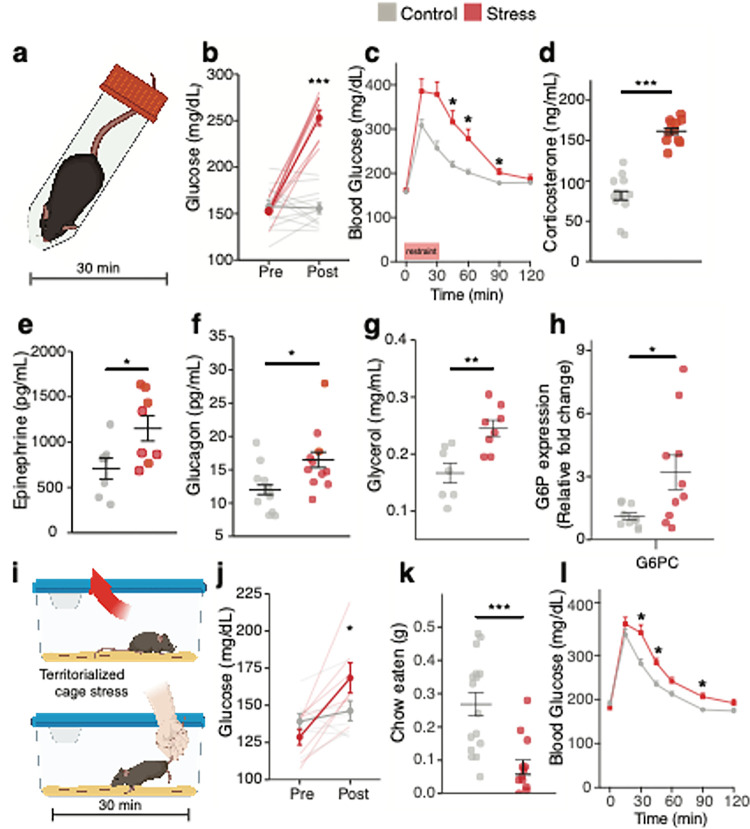
Metabolic effects of acute stressors **a,** Schematic of 30 minutes restraint stressor. **b,** Mean blood glucose pre and post no stress (grey, n = 12) or 30 minutes restraint stress (red, n = 11). *** P < 0.001, control vs. stress. Linear mixed model with post-hoc Tukey’s test. **c,** Effect of restraint stress (30 minutes, red, n = 11) on blood glucose during glucose tolerance testing (GTT) compared to unstressed mice (grey, n = 13), * P < 0.05, control vs. stress, two-way repeated measures ANOVA with post-hoc Sidak’s multiple comparison test (*** P< 0.001). **d-g**, Mean plasma corticosterone (**d**), plasma epinephrine (**e**), plasma glucagon (**f**), and plasma glycerol (**g**) with (red) and without (grey) 30 minutes restraint stress. * P < 0.05, ** P < 0.01, *** P < 0.001, two-tailed t-test, control vs. stress (n = 11/11, n = 6/8 and n = 10/11 respectively). **h,** Relative fold change in liver glucose-6-phosphate expression with (red, n = 10) and without (grey, n = 7) 30 minutes restraint stress. * P < 0.05, two-tailed t-test, control vs. stress. **i,** Schematic of social conspecific odor stressor where male C57Bl6 mice are place in a clean cage or territorialized cage previously occupied by another male mouse for 30 minutes. **j,** Mean blood glucose pre and post clean cage (grey, n = 8) or territorialized cage stress (red, n = 8). * P < 0.05, Linear mixed model with post-hoc Tukey’s test, control vs. stress **k,** Mean food intake during exposure to clean cage (grey, n = 15) or territorialized cage stress (red, n = 12). *** P < 0.001, two-tailed t-test, control vs. stress **l,** Social conspecific odor stress in territorialized cage (red, n = 7) increased blood glucose during glucose tolerance testing (GTT) compared to unstressed mice (grey, n = 7), * P < 0.05, control vs. stress, two-way repeated measures ANOVA with post-hoc Sidak’s multiple comparison test (** P< 0.01). All data represented as mean± SEM. Individual data points represent individual mice.

To investigate the central mechanisms of stress-related metabolic adaptations, we first identified acute stressors that elevated blood glucose levels and suppressed appetite in C57Bl6 mice. Acute restraint stress rapidly elevated blood glucose levels and impaired glucose tolerance in mice that were food-restricted for 6 h prior to testing (Fig. 1a–c). Restraint stress also raised plasma corticosterone (Fig. 1d), consistent with HPA axis activation. Plasma epinephrine, glucagon, and glycerol levels were also elevated by restraint stress (Fig 1e–g), consistent with activation of the adrenomedullary stress system to drive sympathetic input to the adrenal glands, pancreas, and adipose tissue. By contrast, plasma insulin and norepinephrine levels were unaltered by restraint stress in these mice (Fig. S1a,b). Food restriction is known to elicit stress-like metabolic adaptations, including lowered plasma insulin levels and elevated hepatic glucose production^17^. Thus, we assessed metabolic responses to restraint stress in fully fed mice. Acute restraint stress elevated blood glucose (Fig S1c), plasma glucagon (Fig S1d), and corticosterone levels (Fig S1e), and lowered plasma insulin levels (Fig S1f), in the fully fed animals. Restraint stress also increased hepatic expression of glucose-6-phosphatase (Fig 1h) without effects on phosphoenolpyruvate carboxykinase (*Pck1*) (Fig S1g) or liver glycogen content in fed mice (Fig S1h), consistent with stress-induced increases in the capacity of the liver to secrete glucose. Social stress triggered by exposing mice to the odor of a conspecific male in a territorialized cage (Fig 1i) similarly elevated blood glucose, impaired glucose tolerance, and suppressed food intake (Fig. 1j–l). Hence, acute physical and social stressors elicit rapid metabolic adaptations including hyperglycemic and hypophagia.

## Stress activates medial amygdala neurons

**Figure 2:**
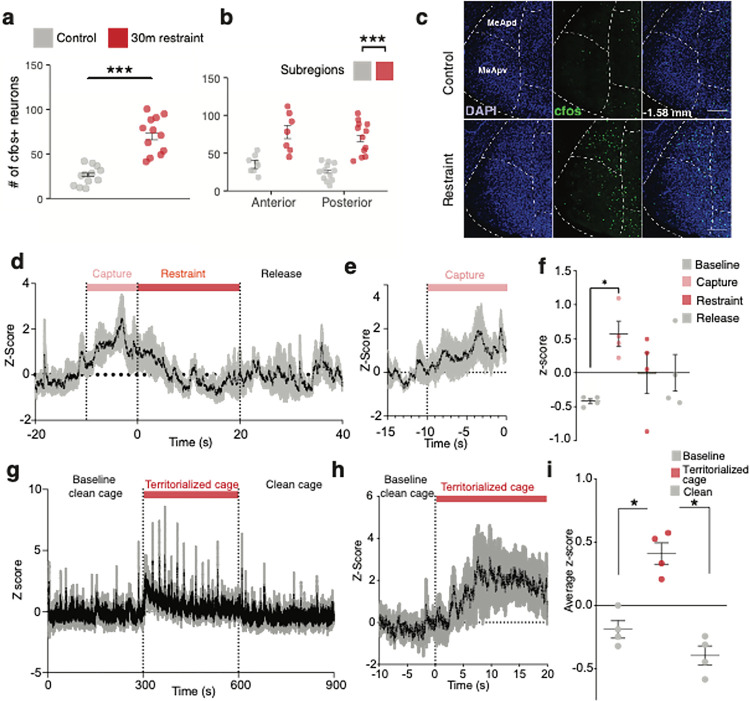
Activation of medial amygdala neurons by acute stressors **a,** Mean number of cfos+ cells across entire medial amygdala (MeA) with (red, n = 12) and without a 30 minutes restraint stressor (grey, n = 12). *** P < 0.001, two-tailed t-test, control vs. stress. **b,** Mean number of cfos+ cells in anterior (between −1.06mm and −1.34mm from bregma) and posterior (between −1.34 and −2.06mm from bregma) MeA with (red) and without a 30 minutes restraint stressor (grey) *** P < 0.001, two-tailed t-test, control vs. stress for anterior and posterior medial amygdala (n = 8/7, 12/12 respectively). **c,** Fos expression in MeA at −1.58 mm from bregma of control (upper panels) and 30 minutes restrained animals (lower panels). MeApd = posterior dorsal subdivision of the MeA; MeApv = posterior ventral subdivision of the MeA; BMA = basomedial amygdala. Scale bar, 200 mm. **d,** MeA GCaMP6m z-score before, during capture and after 20s restraint. Color-coded shade bars indicate periods when the animal was being chased by hand (capture, pink) before restraint (red). n = 4 **e,** Plot of z-score aligned to start of capture period. n = 4 **f,** Mean z-score for baseline, capture, restraint and release periods. * P < 0.05, baseline vs. capture, repeated measures one way ANOVA with post-hoc Sidak’s test, n = 4. **g,** MeA GCaMP6m z-score in clean cage, during 5 minutes exposure to territorialized cage and return to a clean cage. Colour-coded shade bar indicates when the animal was placed in territorialized cage (red). n = 4 **h,** Plot of z-score aligned to placement in territorialized cage. n = 4 **i,** Mean z-score for baseline clean cage, territorialized cage exposure and return to clean cage periods. * P < 0.05, baseline clean cage vs. territorialized cage exposure, territorialized cage exposure vs. return to clean cage, repeated measures one way ANOVA with post-hoc Sidak’s test, n = 4. All data represented as mean± SEM. Individual data points represent individual mice.

Social stimuli are known to modify the activity of medial amygdala (MeA) neurons^18^, and threat-related external and internal sensory signals converge in the MeA^19–21^. MeA neurons send inputs to stress-relevant brain regions that regulate endocrine, autonomic, and metabolic processes, including hypothalamic nuclei^22,23^ and the bed nucleus of the stria terminalis^24^ (BNST). Thus, we hypothesized that MeA neurons participate in metabolic adaptations to acute stress. Restraint stress increased the numbers of cFos immunoreactive (cFos^+^) cells in anterior and posterior domains of the MeA compared to unstressed control mice (Fig 2a–c). Using photometry-based *in vivo* calcium imaging, we found that neural activity was rapidly increased in the MeA during the “capture period” immediately before mice were subjected to restraint stress (Fig 2d–f). MeA activity was suppressed during periods of immobilization in restrained animals (Fig 2d–f) but was markedly increased during the “escape period” when restrained mice were released from immobilization (Fig S2 b-d). Exposing mice to conspecific odor in a territorialized cage also induced a rapid increase in MeA activity (Fig 2g–i). Notably, restraint and social stress-induced increases in MeA activity immediately preceded elevations in blood glucose levels (Fig S2a). By contrast, MeA activity was unrelated to changes in locomotor activity in unstressed conditions, suggesting that MeA neurons do not encode movement *per se* (Fig S2e). These data suggest that neural activity in the MeA is engaged when mice seek to evade or escape threatening stimuli and coincides with the rapid mobilization of energy reserves to support these adaptive behaviors.

## MeA neurons regulate blood glucose levels and feeding behavior

**Figure 3:**
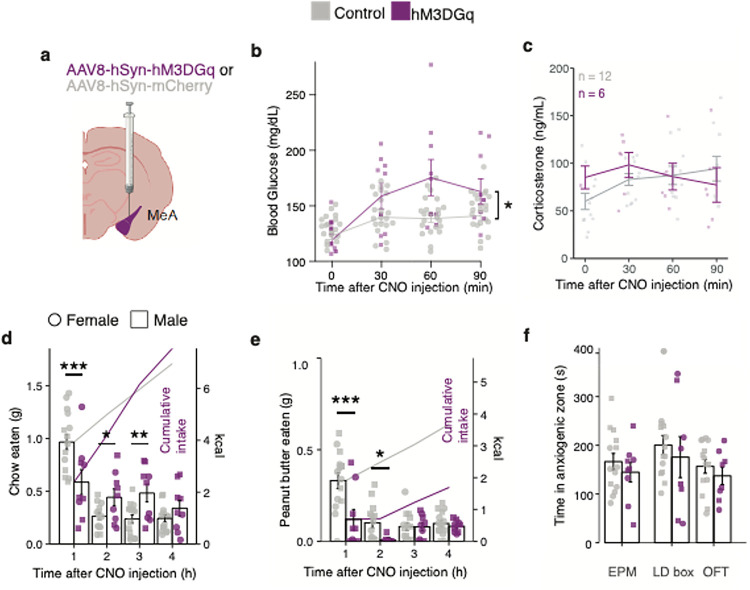
Activation of medial amygdala neurons (MeA) regulates glucose and feeding **a,** Schematic of chemogenetic activation of MeA neurons in vivo. **b,** Blood glucose with CNO injection in 6h fasted mice with MeA expression of hM3DGq (n = 9) compared to those expressing mCherry (n = 20). * P < 0.05, 2 way ANOVA with post-hoc Sidak’s test. **c,** Plasma corticosterone with CNO injection in mice with MeA expression of hM3DGq (n = 12) compared to those expressing mCherry (n = 6). **d-e,** Effects of CNO injection on chow intake in overnight-fasted mice (**d**) and palatable peanut butter intake in fed mice (**e**) expressing hSyn-hM3DGq (n = 9) compared to those expressing hSyn-mCherry (n = 14). Hourly food intake (left axis) and cumulative food intake (right axis) shown. * P < 0.05, ** P < 0.01, control vs. stress *** P < 0.001, hSyn-mCherry vs. hSyn-hM3DGq, linear mixed model with post-hoc Tukey’s test. **f,** Effects of CNO injection on time in the open arms of the elevated plus maze (EPM), light arena in light-dark box (LD box) or center time in the open field test (OFT) in mice expressing hM3DGq (n = 9) compared to those expressing mCherry (n = 14). All data represented as mean± SEM. Individual data points represent individual mice.

Next, we used chemogenetics to investigate MeA involvement in metabolic adaptations to stress. We delivered an adeno-associated virus (AAV) expressing the excitatory hM3Dq DREADD (AAV-hSyn-hM3Gq-mCherry) or a control virus (AAV-hSyn-mCherry) into the MeA of mice (Fig 3a and Fig. S3a). Clozapine-*N*-oxide injection (CNO, 3 mg/kg IP) increased blood glucose levels in DREADD-expressing but not mCherry-expressing control mice (Fig. 3b). Considering that MeA activity was increased by physical and social stressors, we were surprised to find that DREADD-mediated stimulation of MeA neurons elevated blood glucose levels without altering plasma corticosterone (Fig 3c and Fig S3e), insulin, or glucagon (Fig S3c,d). This suggests that MeA neurons precipitated increases in blood glucose levels without recruiting stress-related glucoregulatory hormones from the adrenal cortex or pancreas controlled by the HPA and adrenomedullary stress systems. In addition to elevating blood glucose levels, DREADD-mediated stimulation of the MeA induced stress-like suppression of food intake (standard laboratory chow) in food-deprived mice that had been fasted overnight (Fig 3d). MeA stimulation similarly suppressed reward-driven consumption of palatable food in fully-fed mice (Fig 3e) as well as in food-deprived mice that had been fasted overnight (Fig S3b). By contrast, MeA activation had no effect on fear or anxiety-related defensive behaviors in an elevated plus-maze, light-dark box, or open field apparatus (Fig 3f). Together, these data suggest that MeA neurons are activated by acute stressors to drive metabolic but not behavioral adaptations to stress.

## MeAVMH neurons regulate stress-induced hyperglycemia

**Figure 4:**
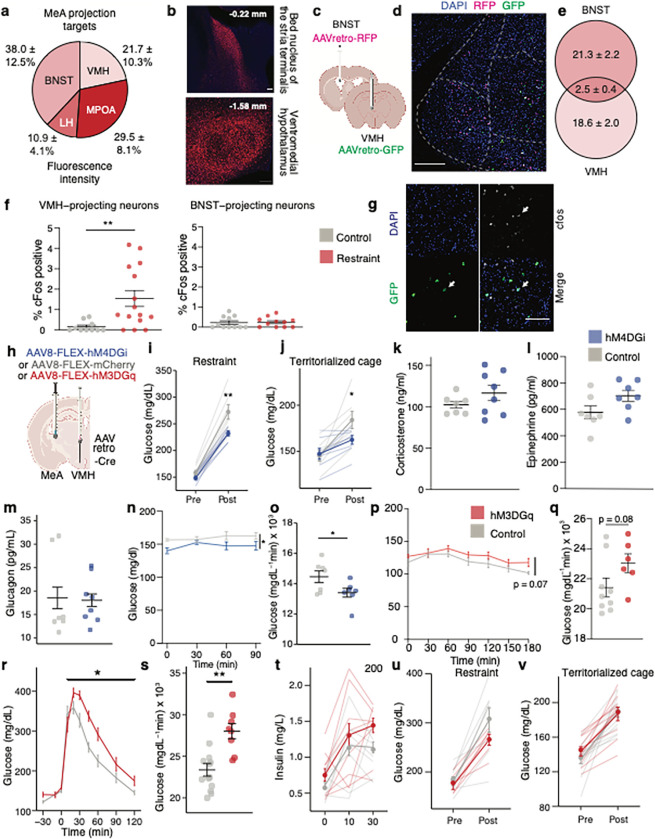
MeA neurons projecting to VMH regulate blood glucose **a, b,** Anterograde tracing from MeA neurons. Quantification (**a**) and expression (**b**) of mCherry-labeled synaptophysin in the BNST (upper panel) and VMH (lower panel), n = 3, scale bar, 100 mm. **c-e,** Retrograde tracing from MeA projection sites in BNST and VMH. c, Schematic of dual retrograde tracing with injection of AAVretro-RFP into BNST and AAVretro-GFP into VMH **d,** RFP (BNST-projecting neurons) and GFP (VMH-projecting neurons) expression in MeA, scale bar, 200 mm. **e,** Quantification of BNST-projecting and VMH-projecting neurons in MeA, n = 3, **f,** Quantification of cfos expression in VMH-projecting (left) and BNST-projecting (right) MeA neurons with (red) and without 30 minutes restraint stress (grey) (MeA-VMH: n = 10 control, n = 15 stress, MeA-BNST, n = 11 control and stress). ** P < 0.01, two-tailed Mann-Whitney, control vs. stress. **g,** Cfos expression in GFP-labeled VMH-projecting neurons in MeA, arrows indicate neurons with co-expression of cfos and GFP, scale bar, 100 mm. **h,** Schema of strategy for chemogenetic modulation of MeA-VMH circuit. AAVretro-cre was delivered into the VMH and cre-dependent Flex-hM4DGi (silencing), Flex-hM3DGq (activating) or Flex-mCherry (control) were delivered into the MeA. **i, j,** Effects of CNO injection on blood glucose in restrained mice (**i**) and mice placed in territorialized cage (**j**) expressing Flex-hM4Gi (n = 8) compared to those expressing Flex-mCherry (n = 7). * P < 0.05, ** P < 0.01, control vs. hM4DGi, linear mixed model with post-hoc Tukey’s test. **k-m,** Effects of CNO injection on plasma corticosterone (**k**), plasma epinephrine (**l**) or plasma glucagon (**m**) in restrained mice expressing Flex-hM4Gi (n = 8) compared to restrained mice expressing Flex-mCherry (n = 7). **n,o,** Effects of CNO injection on blood glucose (**n**) and cumulative change in blood glucose (**o**) in unstressed mice expressing Flex-hM4DGi (n = 8) compared to those expressing Flex-mCherry (n = 7). * P < 0.05, two-way ANOVA with post-hoc Sidak’s test and * P < 0.05, two-tailed t-test, control vs. hM4DGi. **p, q,** Effects of CNO injection on blood glucose (**p**) and cumulative change in blood glucose (**q**) in unstressed mice expressing Flex-hM3DGq (n = 6) compared to those expressing Flex-mCherry (n = 9). **r-t,** Effects of CNO injection on blood glucose (**r**), cumulative change in blood glucose (**s**) and plasma insulin (**t**) during glucose tolerance testing (ipGTT) in unstressed mice expressing Flex-hM3DGq (n = 8) compared to those expressing Flex-mCherry (n = 13 for r and s, n = 8 for t), * P < 0.05, ** P < 0.01, control vs. hM3DGq, linear mixed model with post-hoc Tukey’s test. **u, v,** Effects of CNO injection on blood glucose in restrained mice (**u**) and mice placed in territorialized cage (**v**) expressing Flex-hM3DGq (n = 7 for u, n = 10 for v) compared to those expressing Flex-mCherry (n = 8 for u, n = 13 for v). All data represented as mean± SEM. Individual data points represent individual mice.

We next investigated the circuit-level mechanisms by which MeA neurons elevate blood glucose levels and suppress appetite. Injection of mCherry-tagged synaptophysin (a synaptically localized protein) into the MeA revealed dense mCherry-expressing axon terminals in hypothalamic regions known to regulate metabolism, including the medial preoptic area, lateral hypothalamus, and ventromedial hypothalamus (VMH) (Fig 4a,b). We also detected mCherry-expressing terminals in the BNST, a component of the extended amygdala known to regulate physiological and behavioral adaptations to stress^25^ (Fig 4a,b). Since circuits involving the VMH and BNST contribute to glucose regulation^26,27^ and are engaged by stressful stimuli^25,28^, we investigated whether the MeA neurons that project to the VMH (MeA^VMH^ neurons) and/or BNST (MeA^BNST^ neurons) regulate metabolic adaptations to stress. First, we determined whether the same populations of MeA neurons project to both VMH and BNST. We injected a retrograde-traveling AAV expressing red fluorescent protein (AAVretro-RFP) into the BNST and an AAVretro expressing green fluorescent protein (AAVretro-GFP) into the VMH (Fig 4c). We found that less than 12% of labeled neurons co-expressed both RFP and GFP (Fig 4d,e). This suggests that largely non-overlapping populations of MeA neurons project to VMH or BNST. Next, we used cFos immunolabelling combined with AAVretro tracing to determine whether MeA^VMH^ or MeA^BNST^ neurons are activated by stressors that elevate blood glucose levels. Restraint stress increased numbers of cFos+ MeA^VMH^ neurons by ~2-fold without increasing numbers of cFos+ MeA^BNST^ neurons (Fig 4f,g). This suggests that acute stress preferentially increases the activity of MeA^VMH^ neurons. Therefore, we investigated whether inactivation of the MeA^®^VMH circuit modified metabolic responses to acute stress. Chemogenetic silencing of MeA^VMH^ neurons was achieved by co-injecting AAVretro-Cre into the VMH and AAV-DIO-hSyn-hM4Gi into the MeA of the same mice (Fig 4h). Silencing MeA^VMH^ neurons with CNO (3 mg/kg) blunted the hyperglycemic responses elicited by restraint stress and exposure to a territorialized cage (Fig 4i,j). By contrast, stress-induced increases in corticosterone, epinephrine, and glucagon were unaltered by inactivation of MeA^VMH^ neurons (Fig 4k–m). Silencing MeA^VMH^ neurons elicited a modest decrease in plasma glucose after CNO injection in fasted mice but did not alter levels of plasma insulin or norepinephrine (Fig 4n,o and Fig S4a,b), or alter responses in an glucose tolerance test (GTT) (Fig S4c,d). Silencing MeA^VMH^ neurons also had no effect on food intake in fasted mice (Fig S4e) or anxiety-like behavior in an open-field apparatus (Fig S4f,g). Next, we expressed hM3Dq in MeA^VMH^ neurons (Fig 4h) and assessed blood glucose regulation. In fasted mice, blood glucose tended to be increased by chemogenetic stimulation of MeA^VMH^ neurons (Fig 4p,q). However, stimulating MeA^VMH^ neurons resulted in the persistent elevation of blood glucose levels in an GTT test relative to control mice (Fig 4r,s). This suggests that elevated activity of MeA^VMH^ neurons delays the recovery of blood glucose to homeostatic levels. Hyperglycemia induced by restraint or social stress was not further elevated by DREADD-mediated stimulation of MeA^VMH^ neurons (Fig 4u,v), suggesting that stress-induced increases in MeA activity occluded any further response to their chemogenetic stimulation. Notably, the persistently elevated blood glucose levels during GTT evoked by stimulating MeA^VMH^ neurons occurred without any change in plasma insulin (Fig 4t). Similarly, plasma corticosterone, epinephrine, glucagon levels, and insulin sensitivity (Fig S5b-e) were not altered by MeA^VMH^ activation. However, chemogenetically stimulating MeA^VMH^ neurons elevated blood glucose levels in hypoglycemic animals that had been treated with insulin to lower their baseline glucose levels (Fig S5a). This suggests that MeA^VMH^ neurons are likely not involved in regulating blood glucose homeostasis through mechanisms involving insulin and other classical glucoregulatory hormones but instead contribute to stress-induced glycemic adaptions through an unknown mechanism. Chemogenetic activation of MeA^VMH^ neurons had no effect on food intake in fasted mice (Fig S5h) or anxiety-like behavior (Fig S5f,g), suggesting that MeA^VMH^ neurons are involved specifically in glycemic responses to stressful stimuli. To assess the circuit-level specificity of these findings, we characterized the effects of DREADD-mediated stimulation of MeA^BNST^ neurons. In keeping with the absence of stress-induced increases of cFos in MeA^BNST^ neurons, we found that chemogenetic stimulation of these neurons had no effect on basal glucose levels or changes in a GTT (Fig S6a-c), plasma insulin, glucagon, or corticosterone levels (S6d-f), feeding behavior in fasted mice (S6l), or anxiety-like behavior (Fig S6j,k). Together, these findings suggest that MeA^VMH^ neurons regulate hyperglycemic but not hypophagic or behavioral adaptations to stress through a mechanism independent of major adrenal and pancreatic glucoregulatory hormones.

## MeA neurons control hepatic glucose production

**Figure 5:**
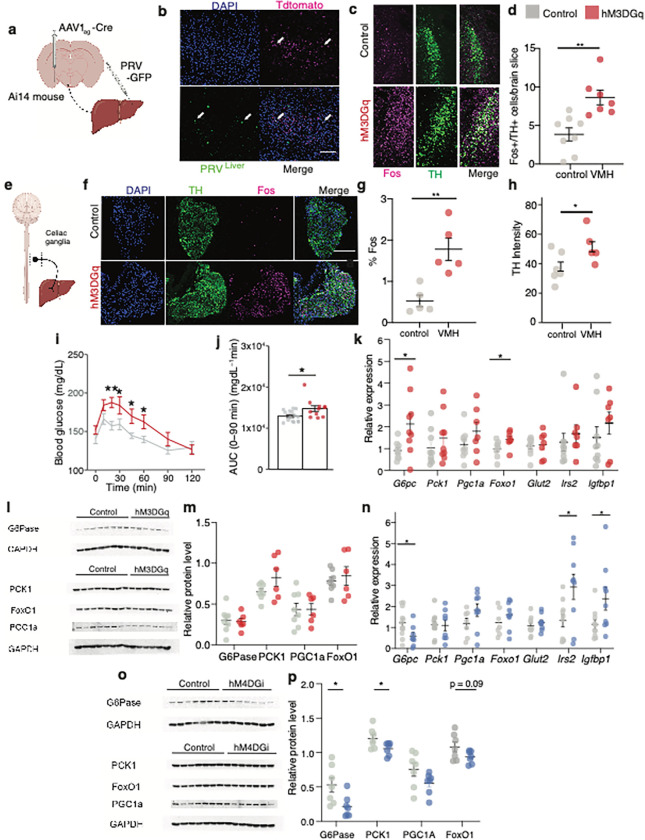
MeA-VMH neurons regulate liver glucose production via sympathetic activationa **a,** Schematic of strategy to trace polysynaptic inputs from MeA neurons to liver using anterograde tracing with AAV1-cre injection into the MeA in an Ai14 tdTomato reporter mice to mark VMH neurons receiving MeA inputs and retrograde tracing with PRV-GFP injection into liver to mark neurons synaptically connected to the liver. **b,** Expression of tdTomato in VMH neurons receiving MeA inputs and PRV-GFP in VMH of Ai14 mice. Arrows mark neurons with colocalization. **c,d,** Cfos expression (**c**) and quantification (**d**) of cfos+ in catecholaminergic (tyrosine hydroxylase, TH+) neurons of the locus coeruleus with CNO treatment in unstressed mice expressing Flex-hM3DGq (n = 7) in MeA-VMH neurons (right) compared to those expressing Flex-mCherry (n = 8) (left).** P < 0.01, two-tailed t-test. **e, f,** Schema of sympathetic outflow to liver via celiac ganglia (**e**), expression of cfos in tyrosine hydroxylase (TH)+ neurons of the celiac ganglia with CNO treatment in unstressed mice expressing Flex-hM3DGq (n = 5) in MeA-VMH neurons (bottom panels) compared to those expressing Flex-mCherry (n = 5) (top panels). **g, h,** Quantification of percentage of celiac ganglia cfos+/TH+ neurons (**g**) and TH intensity (**h**) with CNO treatment in unstressed mice expressing Flex-hM3Dq (n = 5) in MeA-VMH neurons compared to those expressing Flex-mCherry (n = 5), *P< 0.05, ** P < 0.01, two-tailed t-test. **i, j,** Effects of CNO treatment on blood glucose (**i**) and cumulative change in blood glucose (**j**) during pyruvate tolerance testing in unstressed mice expressing Flex-hM3DGq (n = 11) in MeA-VMH neurons compared to those expressing Flex-mCherry (n = 15), *P< 0.05, ** P < 0.01, linear mixed model with post-hoc Tukey’s test. **k,** Effects of CNO treatment on relative hepatic expression of gluconeogenic genes, glucose-6-phosphatase (*G6Pc*), Phosphoenolpyruvate Carboxykinase 1 (*Pck1*), Peroxisome Proliferative Activated Receptor, Gamma, Coactivator 1, Alpha (*Pgc1a*), Forkhead box O1 (*Foxo1*), glucose transporter 2, (*Glut2*), insulin receptor substrate 2 (*Irs2*) and insulin-like growth factor binding protein 1 (*Igfbp1*) in unstressed mice expressing Flex-hM3Dq (n = 9) in MeA-VMH neurons compared to those expressing Flex-mCherry (n = 8) *P< 0.05, Mann-Whitney U test. **l,m,** Western blot of liver protein (**l**) and quantification (**m**) of effects of CNO treatment on protein levels of G6Pase, PCK1, PGC1a and FOXO1 in unstressed mice expressing Flex-hM3Dq (n = 7) in MeA-VMH neurons compared to those expressing Flex-mCherry (n = 7) *P< 0.05, Mann-Whitney U test. **n,** Effect of CNO treatment on relative expression of hepatic *G6pc, Pck1, Pgc1a, Foxo1, Irs2* and *Igfbp1* in stressed mice expressing Flex-hM4DGi (n = 8) in MeA-VMH neurons compared to those expressing Flex-mCherry (n = 7) *P< 0.05, Mann-Whitney U test. **o,p,** Western blood of liver protein (**o**) and quantification (**p**) of effects of CNO treatment on G6PC, PCK1, PGC1a, and FOXO1 protein levels in stressed mice expressing Flex-hM4DGi (n = 7) in MeA-VMH neurons compared to those expressing Flex-mCherry (n = 8) *P< 0.05, Mann-Whitney U test. All data represented as mean± SEM. Individual data points represent individual mice.

We postulated that MeA neurons elevate blood glucose levels by “bypassing” adrenal and pancreatic systems to directly stimulate hepatic glucose production. Such a mechanism would position MeA neurons to rapidly elevate blood glucose levels during stressful events independent of relatively slow-acting glucoregulatory hormones. To investigate this possibility, we first explored whether MeA neurons communicate with the liver through synaptic connections. Thus, we injected AAV1 expressing Cre recombinase into the MeA of Ai14 reporter mice for the anterograde and transsynaptic fluorescent tagging of downstream neurons^29^. In these mice, neurons in the MeA neurons and those that receive synaptic input from the MeA will express the red fluorescent protein tdTomato (tdTom) in response to Cre-mediated recombination events. We injected pseudorabies virus expressing GFP (PRV-GFP) into the liver of these same animals (Fig 5a). As PRV-GFP travels retrogradely from the sites of injection along synaptically connected neurons, this allowed us to map GFP^+^ neurons that provide polysynaptic inputs to the liver (Fig 5a). We detected dual-labelled cells in the VMH that co-expressed both tdTom and GFP (Fig 5b). This suggests that MeA^VMH^ neurons communicate with the liver via networks of polysynaptic connections. We hypothesized that MeA^VMH^ neurons signal to the liver via sympathetic efferent neurons of the autonomic nervous system. Consistent with this possibility, DREADD-mediated activation of MeA^VMH^ neurons increased cFos expression in tyrosine-hydroxylase (TH) expressing neurons in the locus coeruleus (Fig 5c,d) and in TH-positive neurons in the coeliac ganglia (Fig 5e–g), which are major central and peripheral hubs, respectively, of the sympathetic efferent pathway. In addition, activation of MeA^VMH^ neurons increased TH intensity in coeliac ganglia neurons (Fig 5h), an indication of sympathetic neural activation^30^.

Sympathetic activity regulates hepatic glucose production by modulating de novo synthesis of glucose (gluconeogenesis) and the breakdown of glycogen (glycogenolysis)^31^. Gluconeogenesis relies on the conversion of pyruvate to oxaloacetate, and ultimately to glucose^32^. Hence, gluconeogenesis can be assessed by monitoring elevations in blood glucose levels after pyruvate administration. In keeping with MeA regulation of hepatic gluconeogenesis, chemogenetic stimulation of MeA^VMH^ neurons elevated blood glucose in unstressed mice after pyruvate administration (2 g/kg IP) (Fig 5i,j). Next, we assessed the effects of MeA^VMH^ modulation on the expression of glucose-6-phosphatase (*G6pc*) and other stress-sensitive hepatic genes that regulate gluconeogenesis (Fig 1h). Activation of MeA^VMH^ neurons in unstressed mice increased liver expression of G6pc, which controls the final rate-limited step in hepatic gluconeogenesis^33^, as well as *Foxo1*, a transcriptional activator of gluconeogenic genes^34^ (Fig 5k). We did not detect any change in protein levels of G6Pase in unstressed mice at this time point (Fig 5l,m) however G6Pase activity is regulated by glucose-6-phosphate concentrations^35^ as well as by protein levels. Stimulating MeA^VMH^ neurons in unstressed mice also increased liver glycogen content (Fig. S7a), suggesting these neurons do not promote glycogenolysis. Instead, increased glycogen content after MeA^VMH^ neuron activation could reflect the inhibition of glycogenolysis by increased plasma glucose^36^. Consistent with this interpretation, liver glycogen content is known to be increased in mice with increased gluconeogenesis secondary to insulin insensitivity^37^. We next assessed the effects of chemogenetically silencing MeA^VMH^ neurons on the expression of hepatic genes that regulate gluconeogenesis in stressed mice. We found that silencing of MeA^VMH^ neurons blunted the stress-induced increase in hepatic *G6pc* gene expression, increased expression of liver insulin-responsive genes *Irs2* and *Igfbp1* (Fig 5n) and decreased liver G6Pase and PCK1 protein levels (Fig 5o,p), without effects on liver glycogen content (Fig S7b). Together, these findings suggest that MeA^VMH^ neurons regulate stress-induced hyperglycemia by a mechanism involving sympathetic recruitment of hepatic gluconeogenesis.

## Recurrent stress disrupts MeA-liver signaling to precipitate metabolic dysfunction

**Figure 6:**
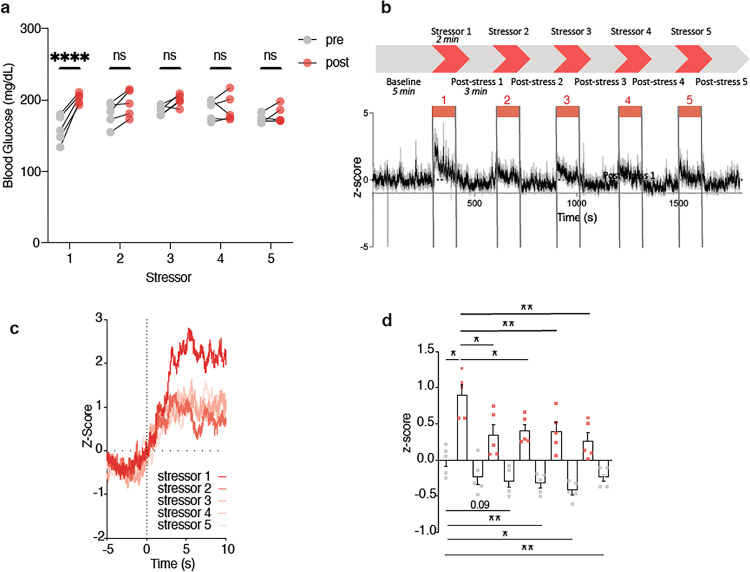
Repeated stress blunts MeA neuron activation **a,** Blood glucose before and after repeated 2 minutes exposure to territorialized cage stress, n = 5, *P< 0.05, Mann-Whitney U test. **b,** MeA GCaMP6m z-score before, during and after repeated 2 minutes exposures to territorialized cage. Colour-coded bars indicate each repeated 2 minutes exposure period (red), n = 5. **c,** Plot of z-score aligned to start of each territorialized cage exposure period (n = 5). **d,** Mean z-score for baseline, each exposure period, each period between exposures and after final exposure. *P< 0.05, ** P < 0.01, repeated measures one way ANOVA with Sidak’s multiple comparison test, n = 5.

Finally, we investigated whether glycemia regulation by MeA neurons was modified with repeated stress exposure. This is important because prolonged stress is known to precipitate metabolic abnormalities including type 2 diabetes (T2D) and obesity^38–41^. We began by using fiber photometry to assess neural activity in the MeA of mice as they were subjected to repeated stress by exposure to a conspecific odor in a territorialized cage. In keeping with our previous results, initial exposure of mice to the territorialized cage (5 min) elicited hyperglycemia but repeated exposure induced rapid habituation in stress-induced hyperglycemia (Fig 6a). We then examined MeA activity in mice that were subjected to repeated 2 min exposures to the territorialized cage followed by intervening periods of recovery in a clean cage (3 min). As expected, MeA activity was markedly increased during the initial period of stress exposure in the otherwise stress-naïve mice (Fig 6b–d). However, the magnitude of stress-induced increases in MeA activity gradually decreased as the mice were subjected to subsequent bouts of stress (Fig 6b–d), in keeping with habituation of the hyperglycemic stress response (Fig. 6a). Notably, MeA activity during the post-stress recovery periods also progressively decreased in these animals such that by the end of testing, MeA activity was lower than pre-stress baseline levels (Fig 6b–d). These data suggest that repeated stress exposure induces counterregulatory adaptations in the activity of the MeA neurons.

**Figure 7:**
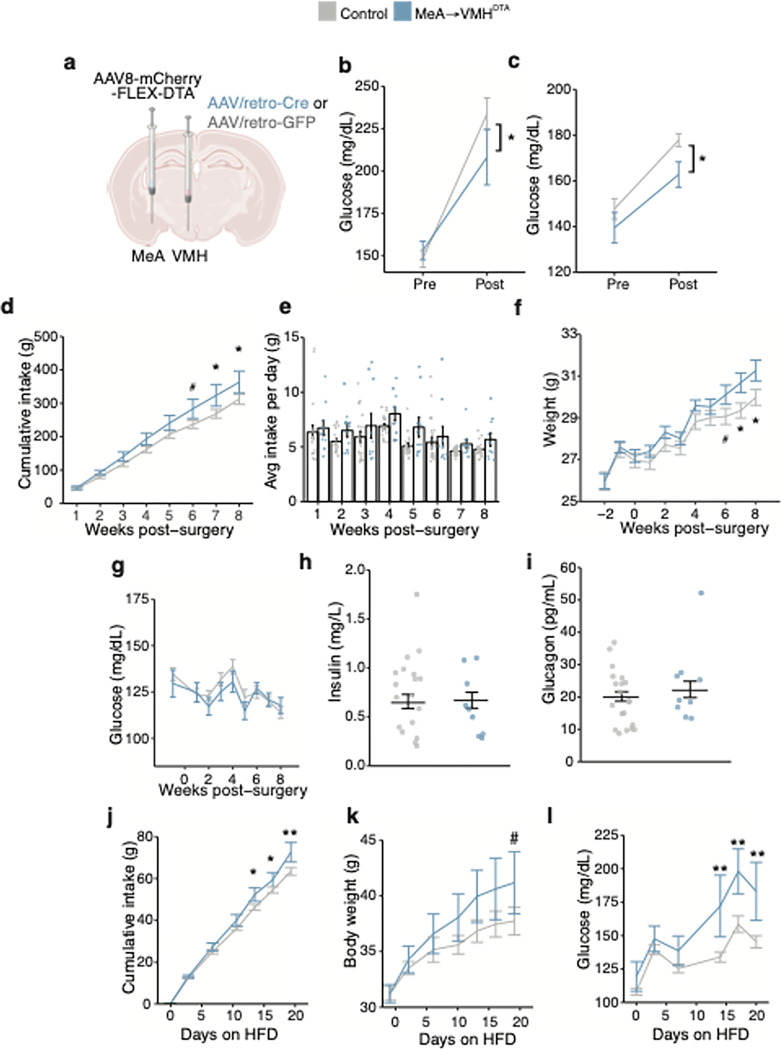
Chronic MeA-VMH neural silencing promotes weight gain and hyperglycemia **a,** Schema of strategy to chronically silence MeA-VMH circuit. AAVretro-Cre was or AAVretro-GFP were delivered into the VMH and Cre-dependent Flex-diphtheria toxin subunit A (DTA) delivered into the MeA. **b-c,** Effect of chronic silencing of MeA-VMH neurons with DTA (n = 10) on blood glucose during restraint stress (**b**) and territorialized cage stress (**c**) compared to control mice (n = 20), *P< 0.05, linear mixed model with post-hoc Tukey’s test. **d-f,** Effect of chronic silencing of MeA-VMH neurons with DTA (n = 17) on cumulative food intake (**d**), daily food intake (**e**) and body weight (**f**) compared to control mice (n = 30), # P < 0.1, *P< 0.05, linear mixed model with post-hoc Tukey’s test. **g-i,** Effect of chronic silencing of MeA-VMH neurons with DTA (n = 9) on random blood glucose (**g**), plasma insulin (**h**) and plasma glucagon (**i**) compared to control mice (n =17). **j-l,** Effect of chronic silencing of MeA-VMH neurons with DTA (n = 9) in combination with a high fat diet on cumulative food intake (**j**), body weight (**k**), and blood glucose (**l**) compared to control mice fed a high fat diet. *P< 0.05, ** P< 0.01, linear mixed model with post-hoc Tukey’s test. All data represented as mean± SEM. Individual data points represent individual mice.

We investigated the functional significance of the suppressant effects of prolonged or repeated stress exposure on MeA activity. To this end, we bilaterally ablated MeA^VMH^ neurons by conditionally expressing diptheria toxin subunit A (DTA) in these neurons (Fig 7a). Insulin sensitivity, and glucose and pyruvate tolerance did not differ between MeA^VMH-DTA^ lesioned and control mice (Fig S7c-e). Similarly, plasma insulin, glucagon, corticosterone, epinephrine, and norepinephrine levels (Fig S7f-j), and behavior in the open-field test (Fig S7k,l) did not differ between MeA^VMH-DTA^ lesioned and control mice. However, mice in which MeA^VMH^ neurons had been ablated demonstrated blunted hyperglycemic responses to restraint and territorialized cage stressors (Fig 7b,c), consistent with the attenuated hyperglycemic response to stress observed in mice after DREADD-mediated silencing of MeA^VMH^ neurons. Strikingly, we found that MeA^VMH-DTA^ lesioned mice consumed greater quantities of food and had elevated body weight relative to control mice (Fig 7d–f) without differences in blood glucose, plasma insulin, or plasma glucagon (Fig 7g–i). Chronic stress is known to increase food intake in rodents and humans, particularly the consumption of energy-dense food items, which is thought to contribute to stress-related weight gain and vulnerability to T2D^41–45^. When given access to high-fat diet, MeA^VMH-DTA^ lesioned mice consumed greater quantities of food, gained more weight, and had significantly higher blood glucose levels than control mice (Fig 7j–l). Hence, these findings suggest that stress-induced deficits in the activity MeA^VMH^ neurons increase vulnerability to metabolic abnormalities in individuals exposed to prolonged periods of stress.

## Summary

Stress elicits highly orchestrated metabolic responses that play a crucial role in supporting the behavioral adaptations to stress that are crucial for survival. The neural mechanisms underlying stress-related metabolic plasticity are largely unknown. Our findings identify a crucial role for a population of hypothalamus-projecting MeA neurons in regulating hyperglycemic responses to physical and social stressors. Unexpectedly, MeA neurons modulated blood glucose independent of classical adrenal and pancreatic glucoregulatory hormones. Instead, MeA neurons provide polysynaptic input to the liver via the sympathetic nervous system to stimulate hepatic gluconeogenesis. Our findings provide compelling evidence that amygdalar circuits orchestrate metabolic responses to stress through the rapid recruitment of liver glucose release. Repeated exposure to stress induced striking adaptations in the activity of these hepato-regulatory MeA neurons, which precipitated persistently elevated blood glucose levels, hyperphagia, and weight gain. If these findings extend to humans, they suggest that dysregulation of MeA signaling contributes to the increased incidence of metabolic dysfunction in those subjected to prolonged periods of stress.

## Methods

### Animals

Mice (8+ weeks old) were housed under controlled light conditions (12 h light/12 h dark) and temperature (22°C) and fed *ad libitum* on standard mouse chow. Mice were randomized to treatment group based on body weight. Unless noted, all animals were male. All mice in functional studies were singly housed to facilitate accurate food intake measurements, except for one cohort of mice in the lesion study, which were kept group housed. All other mice were kept group housed. Mice used were: B6.Cg-Gt(ROSA)26Sortm14(CAG-tdTomato)Hze/J, with Cre-dependent tdTomato (Ai14; Jax# 007914)^46^ and C57BL/6J (Jax# 000664). Animal care and experimental procedures were performed with the approval of the Animal Care and Use Committee of Icahn School of Medicine at Mount Sinai under established guidelines.

### General surgical procedures

All surgeries were performed under aseptic conditions. Mice were anaesthetized using 2% isoflurane and the top of the head was shaved then cleaned with 70% ethanol. Ophthalmic ointment was applied to the eyes and subcutaneous injections of buprenorphine (0.05mg/kg) were given to each animal prior to surgery. An incision was made in the midline and small craniotomies were made using a dental drill. Thirty-three gauge syringe needles (Hamilton) were used to unilaterally or bilaterally infuse virus into the brain at a rate of 0.1 μl/min. The following volumes and coordinates were used: MeA – 0.3–0.5 μl, 1.4 mm posterior, 2.5 mm lateral (2.55 mm if mouse body weight > 25 g, 2.6 mm if mouse body weight > 30 g), and 5.35 mm ventral from bregma; VMH – 0.3 μl, 1.2 mm posterior, 0.23 mm lateral, and 5.6 mm ventral from bregma; BNST – 0.3 μl, 0.2 mm anterior, 0.85 mm lateral, and 4.3 mm ventral from bregma. Viral expression was confirmed after euthanasia using a fluorescent Zeiss Axio Observer Z.1 microscope to visualize fluorophores and confirm targeting. Animals with misplaced injections or without virus expression were not included in the analysis.

Transneuronal circuit analysis was performed using a modified pseudorabies virus (PRV) expressing enhanced green fluorescent protein (GFP) (PRV152). PRV-GFP was injected into the liver of Ai14 mice via a Hamilton syringe (5 × 100 nl, 3.96*10^9 pfu/mL). Seven days after the PRV-GFP injections, mice were sacrificed via perfusion and brains dissected and sectioned to visualize PRV-GFP expression.

### Viral vectors

We used the following viruses: AAV8-hSyn-hM3D(Gq)-mCherry (gift from B. Roth, Addgene viral prep #50474-AAV8; RRID:Addgene_50474); AAV8.2-synapsin-mCherry (Virovek, Hayward, CA); AAV8.2-hEF1a-synaptophysin-mCherry (Massachusetts General Hospital Gene Delivery Technology Core, AAV-RN8, RRID:SCR_012544); AAV/retro-RFP (gift from K. Deisseroth, Addgene viral prep #114472-AAVrg, RRID:Addgene_114472); AAV/retro-GFP (gift from B. Roth, Addgene viral prep #50465-AAVrg, RRID:Addgene_50465); AAV2/retro-CAG-Cre-WPRE (Boston Children’s Hospital Viral Core); AAV8-hSyn-DIO-hM3D(Gq)-mCherry (gift from B. Roth, Addgene viral prep #44361-AAV8; RRID:Addgene_44361)^47^; AAV8-hSyn-DIO-mCherry (gift from B. Roth, Addgene viral prep #50459-AAV8; RRID:Addgene_50459); AAV8-EF1a-mCherry-flex-dtA (Canadian Neurophotonics Platform Viral Vector Core Facility, RRID:SCR_016477,)^48^; AAV1-hSyn-Cre (gift from J.M. Wilson, Addgene viral prep #105553-AAV1, RRID:Addgene_105553); AAV.Syn.GCaMP6m.WPRE.SV40 was a gift from Douglas Kim & GENIE Project (Addgene viral prep # 100841-AAV9; http://n2t.net/addgene:100841 ; RRID:Addgene_100841)^49^; PRV-152 (gift from L. Enquist)^50^.

### Calcium imaging: Stereotaxic injection and fiberoptic cannula implantation

Animals were anesthetized with 2% isoflurane and placed in a stereotaxic head frame (Kopf Instruments). Ophthalmic ointment was applied to the eyes and subcutaneous injections of meloxicam (5 mg/kg) and Enrofloxacin (5mg/kg) were given to each animal prior to surgery. The scalp was shaved and scrubbed with iodine and alcohol and an incision made on the midline. A craniotomy was made using a dental drill (0.5mm) at the following coordinates AP: −1.4mm, ML: +2.5mm, DV: 5.35mm. 300 nl of pAAV9.Syn.GCaMP6m was injected at a rate of 100 nl/min using a 10 μl Hamilton syringe controlled by a micro-injector. The needle remained in the injection site for two minutes following completion of delivery before being raised 0.1 mm for a further two minutes before being completely retracted. A fiberoptic cannula (MFC_400/430–0.66_6mm_MF1.25_FLT) (Doric, Quebec) was implanted 0.2mm dorsal to viral injection during the same surgery and was secured to the skull using dental cement (Pearson Dental, CA) and three screws (Plastics One, TX). Animals were allowed at least 6 weeks for recovery and to facilitate sufficient viral expression prior to any experimental procedures.

### Fiber Photometry

Mice were tethered to a patch cable (Doric Lenses, MFP_400/430/1100–0.57_3m_FCM-M1.25). Calcium signals were collected using the Doric^®^ Fluorescence MiniCube and fiber photometry console at a sampling frequency of 12 kHz. GCaMP calcium signal (465nm) and UV isosbestic signal (405nm) were collected through the same fiber and equalized to record an equivalent signal/noise ratio. Custom-generated MATLAB (Mathwork) scripts were used to down-sample and normalize the fluorescence signal. The 405 isobestic fluorescence signal was filtered using a polyfit regression giving a fitted control (F405c). ΔF/F was calculated by subtracting F405c from the GCaMP fluorescence signal (F405) and then dividing by F405c (F465 – F405c)/F405). A Z-score conversion was used to calculate the deviation of the resulting ΔF/F from the averaged signal of the entire recording session.

#### Restraint stress (manual)

Once animals were tethered to the patch cord they were placed in a clean novel cage with bedding and recording was started. A 1 minute baseline recording was collected prior to the animal being manually restrained for 20 seconds. The animal was then released and an additional 1 minute of calcium activity was recorded.

#### Restraint stress (cone)

Prior to being tethered to the patch cable animals were restrained in a plastic DecapiCone (Braintree Scientific, MA) and placed in a clean novel cage with bedding. A small incision was then placed over the fiber and the patch cord was connected. Calcium transients were recorded for 20 minutes while the animal remain secured in the DecapiCone. At this point an incision was made in the cone to release the animal and recording continued for an additional 15 minutes.

#### Territorialized cage stress

Animals were tethered to a patch cord and were placed in a clean novel cage with bedding and recording started. A 5 minute baseline was collected in the “clean” novel cage before the animal was manually picked up by the base of the tail and placed in a novel territorialized cage that was previously occupied by 5 males for 1 week. The animals remained in this cage while calcium transients were recorded for 2 minutes before the animal was placed back in the previous clean cage for 3 minutes. To examine the effects of repeated stress, this cycle was repeated an additional 4 times with a novel territorialized cage each cycle and the same clean cage.

### *In vivo* behavioral testing

Mice were handled for 5–10 days before experiments. Following stereotaxic surgeries, mice were allowed to recover for 3–6 weeks before the start of testing. Where applicable, clozapine-N-oxide (CNO) (Sigma, NIH) was dissolved in 10% DMSO in saline and delivered at a dose of 3 mg/kg, ip. Investigators were blinded to treatment groups.

#### Restraint stress

Mice were fasted for 6h and then either briefly handled and returned to home cage (controls) or restrained in a 50-mL falcon tube with a hole cut for air at the conical end for 30 minutes. Blood glucose was measured before and after the 30 minutes period. When noted, blood glucose was measured every minute for the first 5 minutes of the stressor to assess the time course of stress-induced hyperglycemia. To measure the hormonal and gene expression responses to stress, mice were rapidly anesthetized with 3% isoflurane and blood collected before being euthanized for tissue collection (e.g. liver) at the end of the restraint period. To measure cfos in the MeA after restraint stress, mice were anesthetized with 3% isoflurane and perfused transcardially with 0.9% saline followed by 10% formalin, and the brain was removed 2h after the start of the restraint. For DREADD modulation studies, CNO was administered 30 minutes before restraint.

#### Territorialized cage stress

Mice were placed in an empty, dirty cage previously occupied by 5 male mice. Blood glucose or food intake was measured before and after the 30 minutes period. For blood glucose measurement, mice were fasted for 6h. When noted, blood glucose was measured every minute for the first 5 minutes of the stressor to assess the time course of stress-induced hyperglycemia. To measure food intake, mice were food deprived overnight before placing in the territorialized cage. For DREADD modulation studies, CNO was administered immediately before the test.

#### Repeated territorialized cage stress

To determine the effects of repeated stress on blood glucose, blood glucose was measured before being placed in an empty, dirty cage previously occupied by 5 male mice. The animals remained in this cage for 5 minutes before blood glucose was measured that the animal was placed back into its home cage for 25 minutes. This cycle was repeated an additional 4 times with a novel territorialized cage each cycle.

#### Food intake studies

Mice were food deprived overnight, food deprived for 6 hours in the light phase or allowed to eat *ad libitum*. Food, either in the form of standard rodent chow or palatable food (peanut butter) was then provided in excess, and consumption of food was measured every hour. For DREADD modulation studies, CNO was administered immediately before food was provided.

#### Metabolic studies

For baseline glucose measurements after DREADD modulation, mice were fasted for 6h, and tail vein samples for blood glucose were taken at 0, 30, 60, 90 minutes after i.p. injection of CNO. Later time points were measured in MeA^VMH^ activation studies (120, 150, and 180 minutes after i.p. injection of CNO). To measure tolerance to a glucose challenge, mice were fasted for 6h and tail vein samples for blood glucose were taken at 0, 10, 20, 30, 45, 60, 90, and 120 minutes after injection of glucose (2g/kg body weight). When noted, additional blood was collected at 0, 10, 30, 60, and 90 minutes after glucose injection to measure plasma insulin and glucagon. To measure insulin sensitivity and tolerance to an insulin challenge, mice were fasted for 4h and tail vein samples for blood glucose were taken at 0, 30, 60, 90, and 105 minutes after injection of insulin (0.4–0.6 U/kg body weight, Humulin R HI-210). To measure gluconeogenic capacity, mice were fasted for 4h and tail vein samples for blood glucose were taken at 0, 30, 45, 60, 90, and 105 minutes after i.p. injection of pyruvate (2g/kg body weight, Sigma #P5280). For all metabolic challenges, CNO was injected 30 minutes before the challenge/timepoint 0.

#### Open field activity

Locomotion and anxiety-like behavior were measured in either a clear plexiglass 40 × 40 × 30-cm open field arena using Fusion Software (v5.0) (Omnitech Electronics) or a white acrylic 18 × 18 × 18 in arena using Ethovision XT (Noldus Information Technology Inc., Leesburg, VA) to quantify behavior. Distance traveled and time spent in the center of the open field arena were measured. The test lasted 10 minutes. For DREADD modulation studies, CNO was administered 60 minutes before the start of the test.

#### Elevated plus maze and light-dark box

The light-dark box test was performed on the same 40 × 40 × 30-cm arena as the open field, except a black box was placed on the half the arena to shield it from light. The mice were placed in the light portion and tracked using Fusion Software. For the elevated plus maze, the mice were placed on an open arm of an elevated four-arm maze in which two arms are open and two are enclosed. They were tracked using Ethovision software and total time spent in the open arm was measured. Both tests lasted 10 minutes and CNO was administered 60 minutes before the start of the test.

#### High fat diet

Mice with MeA^VMH^ expression of dtA or GFP were fed a high fat diet (Research Diets, D12492, 60% fat). Food intake, body weight and blood glucose were measured every 3–7 days for 20 days.

### Tissue processing

Blood glucose was determined using a Contour or Contour Next EZ glucometer (Bayer; Leverkusen, Germany). Blood was collected in an EDTA-coated tube (Sarstedt Microvette CB 300 K2E 16.444.100), spun for 10 minutes at 2,000 rpm, 4°C then plasma was separated and stored at −80°C until assay. Liver was flash frozen in liquid nitrogen for gene expression analysis, glycogen and protein analyses, aliquoted and stored at −80°C until processing. Plasma levels of insulin (Mercodia #10–1247-01), glucagon (Crystal Chem #81518), corticosterone (Crystal Chem #80556), epinephrine and norepinephrine (Abnova #KA1877) were determined by ELISA. Liver glycogen was determined by colorimetric assay (Abcam #ab169558). Circulating glycerol and triglyceride levels were measured by enzymatic assay (Sigma-Aldrich # TR0100).

### Western blot

Liver tissue (~20mg) was lysed in 600μl buffer (20 mM Tris, pH 7.4, 150 mM NaCl, 2% Nonidet P-40, 1 mM EDTA, pH 8.0, 10% glycerol, 0.5% sodium deoxycholate, 0.2% semi-dehydroascorbate) supplemented with halt protease and phosphatase inhibitor (Cell Signaling). Livers were first homogenized using Beadbug (Benchmark) and lysates were further sonicated in ice-cold water for 5 min. After centrifuge at 14,000 rpm at 4°C for 10min, the protein supernatants were transferred to a new tube and protein concentration was determined by BCA protein assay kit (Pierce). 30 μg protein was mixed with 6x SDS sample buffer (#BP-11R, Boston BioProducts) and boiled for 5min before loading on SDS-PAGE. Biorad wet transfer system was used. PVDF membranes were blocked with 5% dry milk in TBST (TBS + 0.05% Tween 20) further incubated with primary antibodies (dilute in TBST with 3% BSA+0.05% NaN_3_) at 4°C overnight. PCK1 (#ab70358), PGC1a (#ab54481), FoxO1 (#ab39670), GAPDH (#ab9485) antibodies were from Abcam. G6PC(#NBP1–80533) was from Novus Biologicals. The membrane was washed 4x in TBST with shaking for 10 minutes prior to incubation with secondary antibodies (dilution 1:10,000 in TBST) for additional 2 hr at room temperature (RT). Immune complexes were washed 4x in TBST with shaking for 10 minutes at RT. Membranes were further reacted with Pierce^™^ ECL western blotting substrate and imaged with iBright CL1500 (Thermofisher). Western blot was quantified by Image J.

### Quantitative PCR

Total RNA was extracted from tissue by homogenization in Trizol (Invitrogen) followed by chloroform (Sigma) extraction and isolation using the RNeasy Plus Mini (Qiagen) kit according to manufacturer’s instructions. Complimentary DNA was prepared by reverse transcription of 500 ng total RNA using qScript cDNA SuperMix (Quantabio). The resulting cDNAs were diluted 1: 10 then amplified by real-time PCR using the SYBR green system (Applied Biosystems) according to the manufacturer’s protocols. All mRNA expression data were normalized to *rpl23* expression in the corresponding sample. Fold change in mRNA expression was calculated using the delta-delta Ct method^51^. The follow primers were used: *PCK1* forward – GCGAGTCTGTCAGTTCAATACC, reverse – GGATGTCGGAAGAGGACTTTG; *G6Pc* forward – GGAGGCTGGCATTGTAGATG, reverse – TCTACCTTGCTGCTCACTTTC; *Rpl23* forward – ACTTCCTTTCTGACCCTTTCC, reverse – TTAGCTCCTGTGTTGTCTGC; PGC1a forward – TGAGGACCGCTAGCAAGTTT, reverse – TGTAGCGACCAATCGGAAAT; *FoxO1* forward – GCGTGCCCTACTTCAAGGATAA, reverse – TCCAGTTCCTTCATTCTGCACT; *Glut2* forward – GTTGGAAGAGGAAGTCAGGGCA, reverse – ATCACGGAGACCTTCTGCTCAG; *IRS2* forward – CCAGTAAACGGAGGTGGCTACA, reverse – CCATAGACAGCTTGGAGCCACA; *IGFBP1* forward – GCCCAACAGAAAGCAGGAGATG, reverse – GTAGACACACCAGCAGAGTCCA.

### Immunohistochemistry

#### Brains

The brains of perfused mice were post-fixed in 4% paraformaldehyde at 4°C overnight. 50 mm coronal slices were cut by vibratome (Leica VT1000). For cfos staining, slices were incubated in blocking solution overnight at 4°C (3% normal donkey serum [NDS, Sigma] in 0.01% Triton-X in 0.01M PBS [PBT]) and then in primary antibody in blocking solution at 4°C. The following primary antibodies, concentrations, and incubation periods were used: Cell Signaling rabbit monoclonal anti-cfos (#2250) – 1:500 for 72h; abcam chicken polyclonal anti-mCherry (#ab205402) – 1:1000 overnight or with abcam chicken polyclonal to tyrosine hydroxylase [#ab76442] – 1:500 for 72 hrs. The slices were then washed in 0.01M PBS (3 × 1h), incubated in secondary antibody in blocking solution for 2h at RT, and washed in PBS (2 × 1h), with a final wash overnight at 4°C. The following secondary antibodies and concentrations were used: Jackson Alexa Fluor 647 AffiniPure donkey anti-rabbit (#711–605-152) – 1:250; Jackson Alexa Fluor^®^ 594 AffiniPure donkey anti-chicken (#703–585-155) – 1:2000.

For RFP/mCherry staining to enhance endogenous fluorescence of AAV/retro-RFP, AAV8-hSyn-DIO-hM3D(Gq)-mCherry, and AAV8.2-hEF1a-synaptophysin-mCherry, slices were washed in 0.01M PBS (3 × 10 minutes), incubated in blocking solution (3% NDS in 0.01% PBT) for 1h at RT, incubated in primary antibody (Rockland rabbit polyclonal anti-RFP [#600–401-379] –1:1000) overnight at 4°C, washed in 0.01% PBT (3 × 10 minutes), incubated in secondary antibody (Invitrogen donkey anti-rabbit Alexa Fluor 594 [#A-21207] – 1:500), and washed in PBS (3 × 10 minutes). For GFP staining to enhance endogenous fluorescence of AAV/retro-GFP or PRV-GFP, slices were washed in 0.01M PBS (3 × 10 minutes), incubated in blocking solution (3% NDS in 0.01% PBT) for 1h at RT, incubated in primary antibody (Abcam goat polyclonal anti-GFP (#ab5450) –1:1000) overnight at 4°C, washed in 0.01% PBT (3 × 10 minutes), incubated in secondary antibody (Invitrogen donkey anti-goat Alexa Fluor 488 #A-11055 – 1:500), and washed in PBS (3 × 10 minutes).

#### Celiac ganglia

The celiac ganglia were dissected from MeAàVMH^hM3Dq^ or mCherry control mice euthanized 2h after CNO administration. The tissue was post-fixed in 4% paraformaldehyde at 4°C overnight, cryo-protected in 30% sucrose (Sigma-Aldrich, 50389) in PBS, embedded in O.C.T Compound (Thermofisher Scientific, Watham, MA; 23–730-572), frozen at −80°C, and sectioned at 10μm thickness. Slides were washed in 0.03% PBT (3 × 5 minutes), incubated in blocking solution overnight at 4°C (2% normal donkey serum, 3% bovine serum albumin in 0.03% PBT), incubated in primary antibodies for 48h at 4°C (Cell Signaling anti-cfos – 1:100; abcam chicken polyclonal to tyrosine hydroxylase [#ab76442] – 1:500), washed in 0.03% PBT (3 × 5 minutes), incubated in secondary antibodies for 2h at RT (Jackson AF-647 donkey anti-rabbit – 1:250; Jackson AF-594 donkey anti-chicken – 1:500), and washed in 0.03% PBT (3 × 5 minutes). After staining, tissue sections were mounted with DAPI counterstain (Fluoromount).

### Image quantification

All confocal images were taken at 20X and tiled. All image analyses were performed using FIJI. Investigators were blinded to treatment groups for cfos analyses.

#### Synaptophysin-mCherry

Four weeks after stereotactic surgery, mice were perfused and brains were sliced and stained to enhance mCherry staining. Confocal images were then taken using a Zeiss LSM 780 confocal microscope. Regions of interest (ROI) were drawn based on DAPI staining and the Franklin and Paxinos mouse brain atlas^52^. The same selection was used for each brain region to normalize for area analyzed and fluorescence intensity was measured within the ROI. Values are reported as median pixel intensity ± standard error of the median.

#### Cfos in the brain

Z-stack confocal images were taken using an upright Zeiss LSM 900 (restraint vs. control). To measure cfos expression after stress, an ROI was drawn around the MeA complex, including the dorsal, ventral, and basomedial subregions. Images were made binary and cell quantification was performed using the ‘analyze particle’ function. The JaCOP plugin^53^ was used to measure total expression of cfos after restraint stress or control, overlap of cfos with AAV/retro-RFP (BNST-projecting neurons) and AAV/retro-GFP (VMH-projecting neurons) and overlap of cfos with tyrosine hydroxylase in the locus coeruleus.

#### Cfos in the celiac ganglia

Z-stack confocal images were taken using a Zeiss LSM 900. Tyrosine hydroxylase (TH) expression was used as a mask to select an ROI of only neurons. Then overlap of cfos and DAPI was measured using the JaCOP plugin. Data is reported as number of cfos-positive DAPI particles.

### Quantification and statistical analysis

All data are presented as mean ± SEM unless otherwise indicated. No statistical methods were used to pre-determine sample sizes but our sample sizes are similar to those reported in previous publications.

Injection sites were visualized and verified following behavioral experiments. Animals were excluded for virus expression outside of the MeA or for insufficient virus expression within the MeA. All mice in the Cre-independent DREADD activation experiment (Fig. 2C–I) showed viral spread into the LH; data shown is from DREADD animals with > 60% virus expression in the MeA.

Analyses were performed in RStudio or with Prism (Graphpad, version 9.4.1). Analyses in R were performed with R 3.6 using the lme4, lmerTest, emmeans, and car packages^54,55^. If the total number of data points for an experiment was less than 30, the data was tested for normality using the Shapiro-Wilk test. If the data was normally distributed or n > 30, data were analyzed with statistics were performed using Student’s unpaired two-tailed t-test for comparison between 2 groups, and One-Way Analysis of Variance (ANOVA) with Tukey’s post-hoc HSD for comparison between multiple groups. Repeated studies were examined using a generalized linear mixed model with mouse identity as a random effect to account for repeated sampling across time or two-way repeated measures ANOVA. Cohort was included as a fixed variable where applicable. *P* values were adjusted using post-hoc testing (e.g. Tukey or Sidak’s testing) for multiple comparisons. If the data was not normally distributed, it was analyzed with the Mann-Whitney U test or Kruskall-Wallis rank sum test with Dunn’s *post hoc* tests. Outliers were defined as values 2 standard deviation above or below the mean per group per time point (where applicable) and removed from analyses. P-values <0.05 were considered to be significant.
